# The prevalence of patellofemoral pain in the Rugby League World Cup (RLWC) 2021 spectators: A protocol of a cross-sectional study

**DOI:** 10.1371/journal.pone.0260541

**Published:** 2021-11-24

**Authors:** María B. Sánchez, James Selfe, Michael J. Callaghan

**Affiliations:** Health Professions Department, Manchester Metropolitan University, Manchester, United Kingdom; Istanbul University Istanbul Faculty of Medicine: Istanbul Universitesi Istanbul Tip Fakultesi, TURKEY

## Abstract

Patellofemoral pain (PFP) can cause significant pain leading to limitations in societal participation and physical activity. PFP is usually associated with athletes undergoing intensive physical training, or military recruits; but recent evidence shows that PFP is common in the general population. The relationship of PFP with physical activity is not entirely clear. Our aim is to provide a better estimate of the general population prevalence of PFP and to relate this to the level of physical activity, and demographic characteristics. The Survey instrument for Natural history, Aetiology and Prevalence of Patellofemoral pain Studies (SNAPPS) was developed as a PFP screening tool to be used in the community. The electronic version of the SNAPPS (eSNAPPS) has recently been validated and was used to survey attendees at mass-participation running events. We will use an electronic survey to collect data from a sample of 1100 Rugby League World Cup spectators. The survey will have four sections: i) general and demographic; ii) knee pain (eSNAPPS); iii) level of physical activity; and iv) quality of life in relation to knee pain. The primary analytic approach will be descriptive of PFP prevalence. Secondary analyses will explore the relationships of the presence of PFP and the other variables. We will disseminate this work by publication of peer-reviewed papers in scientific journals, presentations at scientific conferences, and on the dedicated SNAPPS website https://www.snappspfp.com/.

## Introduction

Patellofemoral pain (PFP) is reported to be a common condition that causes significant pain around the knee joint and patella area that mostly affects young adults, limiting their physical activity and social participation, with 74% reducing or stopping sport participation due to symptoms [1]. Prevalence estimates of PFP vary widely and range between 15% and 45% [2, 3]; but the annual incidence and accurate prevalence of PFP are unknown.

The pathology of this disorder is not clearly understood and numerous tissue based structural and clinical models to explain the disorder have been developed [4]. The structures most likely to generate patellofemoral pain are the anterior synovium, infrapatellar fat pad, subchondral bone, and medial or lateral retinaculae [5]. Stress on any or all of these patellofemoral joint structures may lead to pain, and is usually aggravated by at least one activity that loads the patellofemoral joint during weight bearing on a flexed knee. Previously, it was thought that PFP was a condition affecting athletes undergoing intensive physical training, or military recruits; but there is growing evidence that PFP is common in the general population [2]. However, the relationship of PFP with physical activity is not entirely clear. Some authors report that knee problems in young adults appeared to be associated with high levels of physical activity [6], while others have found that physical activity may not have a detrimental effect on the knee joint but may be beneficial to joint health [7].

The diagnosis of PFP has historically been based on a detailed subjective and objective clinical assessment, with pain on a number of special tests including the patellofemoral compression test, palpation of the patella and pain on resisted extension [3]. However, PFP tends to be diagnosed by first excluding other knee conditions [8], such as traumatic knee injuries, and overuse injuries including, iliotibial band friction syndrome and jumper’s knee [9, 10]. Clinical assessment in large cohort studies are costly and inconvenient to participants [8], making the diagnosis and identification of the true prevalence a more complicated task. Dey et al [8] developed and validated a self-reported questionnaire [Survey instrument for Natural history, Aetiology and Prevalence of Patellofemoral pain Studies, SNAPPS, https://www.snappspfp.com/questionnaire) to overcome these limitations and to identify people with PFP between the ages of 18 and 40 in the community and distinguish them from those suffering from other knee (pain) (OKP) conditions. This instrument was validated against the reference standard of clinical testing for PFP diagnosis showing good agreement with a Cohens Kappa of 0.74 (95% CI 0.52–0.91) [8]. SNAPPS has been translated into 10 other languages (https://www.snappspfp.com/questionnaire) and used to collect data in a number of international studies. Recently an electronic version of the SNAPPS tool (eSNAPPS), has also been developed and has been validated (ICC: 0.99, <0.0001) [11]. Yusuf et al. [11] used the eSNAPPS to estimate the prevalence of PFP within individuals who attended mass-participation running events. This study [11] collected 1080 responses from an opportunistic sample, but did not include information about each participant’s level and type of physical activity that could help clarify the PFP-physical activity relationship.

Yusuf et al.’s study [11] was the largest study in UK evaluating the occurrence of PFP within a sample of general population and the second largest in the world. This study, however, was based on an opportunistic sample, making the characteristics of those participants inappropriate for the generalisation of the findings [11]. Furthermore, age and sex of the participants were the only demographic data collected, which represents an important limitation. To obtain a more accurate picture of the prevalence of PFP, other demographic characteristics that might relate to the presence or not of OKP and specifically of PFP need to be recorded.

The study with the largest sample was completed by Xu et al. [12] with 1153 participants. This study assessed the prevalence of PFP in the general population of Chinese young adults (aged 18–40 years) using the SNAPPS as a self-report questionnaire. The authors reported the difference in the prevalence of PFP and other knee pain conditions in males and females, and also in relation to small age group ranges (< 20, 20–30, and 31–40 years) as well as body mass index (< 18.5, 18.5–24, and > 24). Nevertheless, Xu et al. [12] did not include questions to classify the level of physical activity of the participants.

Different tools have been used to measure physical activity and sedentary behaviour in the UK [13]. As part of the Health Survey for England, 2018 [14] information about the level of physical activity of adults was collected using the International Physical Activity Questionnaire (IPAQ). The IPAQ defines activity levels based on the self-reported amount of physical activity performed in a week: people who do less than 30 minutes of moderate or vigorous activity a week are classified as “inactive”, whereas people who do more than 30 minutes of moderate or vigorous activity a week are classified as “active”. In 2018, it was reported that 27% of adults in England were classified as “inactive” [14]. The IPAQ questionnaire, both in short and long forms, has shown very good repeatability (Spearman correlation coefficient 0.8) and a fair to moderate agreement when compared against accelerometers (p = 0.3, 95% CI 0.23–0.36) [15]. Although there is a clear relationship between low levels of physical activity and cardiovascular diseases, there is still no clarity about the direct relationship between the level of physical activity and the presence of PFP/OKP.

Since 2013 the Rugby League World Cup (RLWC) has been taking place every four years, lasting about one month including all the tournaments (men, women and wheelchair). Since it became a regular tournament, an average of 400,000 people have attended each event. The RLWC 2021 will happen in England, and it is expected to be attended by people from many different countries. Despite the tournament organisers optimism, the tournament had to be postponed for a year and will now be staged at the end of 2022. As with any multinational event, we are anticipating that the fans attending the event will have a varied background (e.g. diverse ethnicity, demographics, physical activity level). The RLWC-2021 is therefore a unique opportunity to collect a large data set that will help generate a more accurate picture of the PFP prevalence in the general population.

### Aims and objectives

The main aim of this project, is to provide a more accurate representation of the prevalence of PFP/OKP in the general population and to relate this to the level of physical activity, ethnicity, occupation and socioeconomic status. Thus, the specific objectives of the project are:

1. to determine the prevalence of PFP/OKP in adult spectators (between 18 and 40 years old) attending the RLWC (estimated as the number of individuals with PFP/OKP divided by the total number of surveyed individuals);
2. to determine the relationship between PFP/OKP, or absence of knee pain and the levels of physical activity of the spectators (correlation coefficient of each pain group and the levels of physical activity);
3. to identify the relationship of PFP/OKP, or absence of knee pain and different demographic characteristics (correlation coefficient of each pain group and each of the demographic characteristics);
4. to describe the relationship of PFP/OKP and the quality of life of the spectators (correlation coefficient of each pain group and the quality of life normalised score).

## Materials and methods

### Ethics

Ethical approval for this project has been obtained from the Faculty Research Ethics and Governance Committee (FREGC) of the Manchester Metropolitan University (Ref. 32732, approved 21/04/2021).

All permissions and clearances will be obtained from the event organisers and any other relevant authorities prior to data collection.

All the face-to-face data collection activities will be performed following relevant government guidance on Covid-19 in force at the time. We will use appropriate PPE if government guidelines require this.

### Study design and population

#### Design and sample size

This cross-sectional study will collect data from a minimum sample of 1100 people attending the RLWC-2021. Previous studies with a smaller sample size have suggested that between 17% and 23% of the population in the UK aged 18 to 40 have PFP using the SNAPPS survey [8, 11]. For an estimate of 23%, the more conservative estimate, we would need at least 1100 participants for a precision of ± 2.5% with 95% confidence [16].

#### Settings, participants and eligibility

This project will be conducted face-to-face using an electronic survey completed using hand-held tablets.

Potential participants will be spectators at matches before they enter the main venue prior to kick off. These participants will be identified by the research team who will be at the venues, roving at queues for turnstiles and at food/drink outlets, or at a project tent. Based on previous experiences, we will target different fixtures in separate venues mainly in the Northwest of England. These could be: Anfield, Liverpool (capacity 53,000); Bolton Stadium (capacity 28,000); Old Trafford, Manchester (capacity 76,000). We will be following all UK government coronavirus guidelines at the time of the event, and data collection activities will be adjusted accordingly.

Participants will be included in the study if they are between 18–40 years old, have the capacity to consent, and can read and understand English.

### Data collection tool

Data collection for the project will be carried out using an electronic survey developed using the Jisc Online Survey platform, presented using hand-held tablets. This online survey requires around 10 minutes to be completed.

The following are the sections of the survey:

1. general and demographic information will be recorded to obtain an accurate picture of the PFP presence and distribution in RLWC-2021 spectators (S1 File);
2. information about the level of physical activity and participation in sport and exercise events will be collected using the IPAQ (short);
3. knee pain information, recorded using eSNAPPS, which asks questions on clinical features, pain on activity, and pain location;
4. additional questions about quality of life will be asked in relation to the presence of knee pain (S2 File).

**Fig 1:**
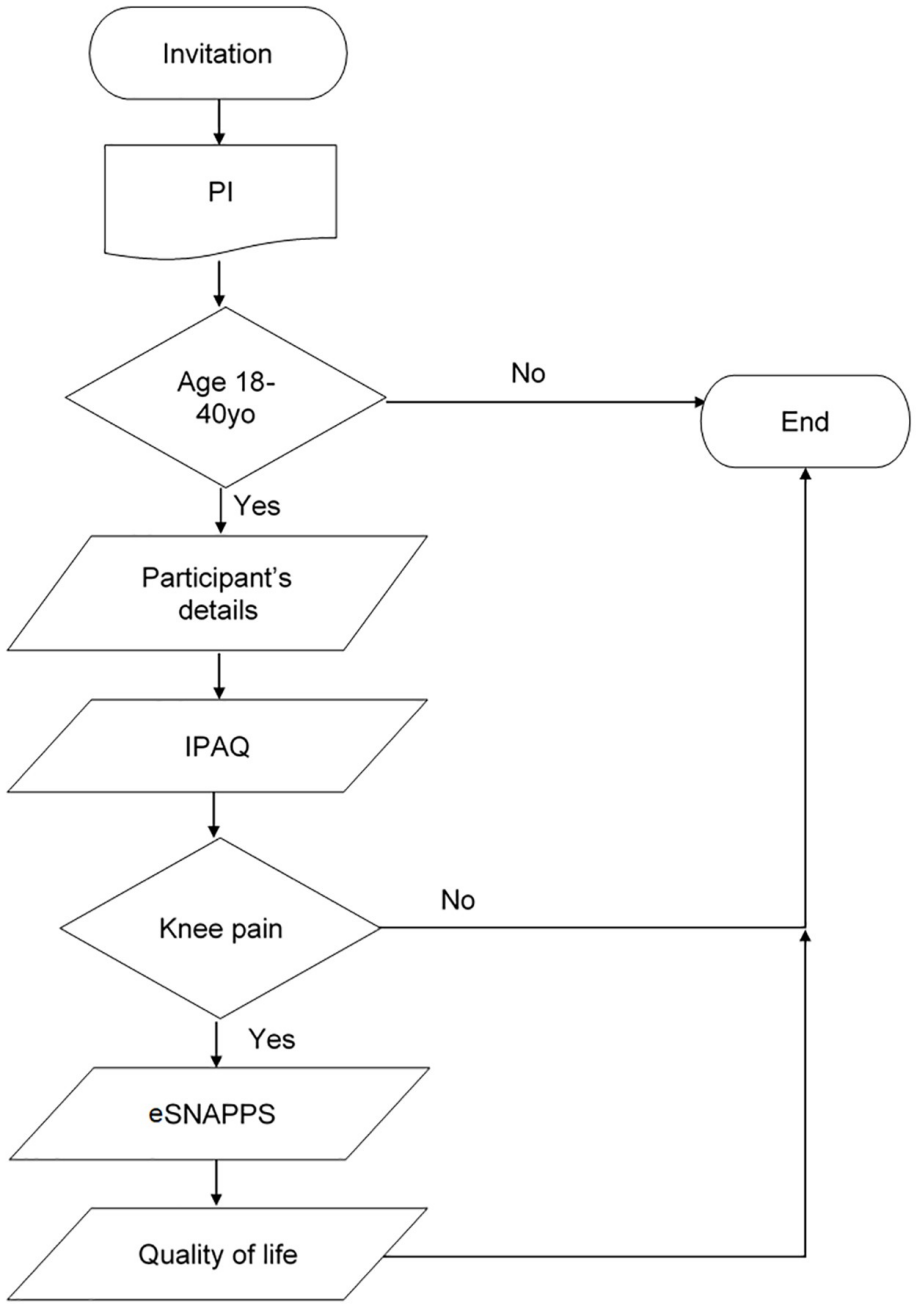
Data collection flow chart. Showing the process for data collection. Acronyms included in the figure: Participant information (PI); International Physical Activity Questionnaire (IPAQ); eSurvey instrument for Natural history, Aetiology and Prevalence of Patellofemoral pain Studies (eSNAPPS).

We will get demographic information and details about the level of physical activity from all the participants. Only participants with knee pain will be asked to complete the eSNAPPS questionnaire and answer the questions about quality of life (Fig 1).

### Data analysis and management

Specific analyses will be performed to satisfy each of the defined objectives.

Firstly, the data about PFP/OKP or absence of knee pain will be analysed considering the following sub-groups: sex, age, body mass index (BMI), level of education, occupation, and socioeconomic status. PFP/OKP will be estimated as the number of individuals with PFP/OKP divided by the total number of surveyed individuals. If the characteristics/numbers of the collected sample allow it, a further analysis looking at the differences in relation to the country of residence of the participants will be included.

Data about PFP/OKP or absence of knee pain will be further analysed as a coefficient correlation in relation to the level of physical activity obtained from the IPAQ scoring. A final analysis will be performed to assess the effect that PFP/OKP has on quality of life; this will be done as a correlation coefficient of each pain group and the quality of life normalised score.

All anonymised data will be uploaded to a cloud-based server for storage. Access to this data repository will be password protected.

Data collected from the survey will be stored on Jisc Online cloud-based servers; this provides data security, minimises storage and transportation considerations and allows easy access. Additionally, data from eSNAPPS does not require data to be entered and processed manually. After data collection, data from the entire survey is retrieved from Jisc Online and downloaded as an Excel file (Microsoft Office, 2016). Data analysis will be performed using a customised R script, which enables automated analysis of large amounts of data.

No identifiable personal data will be collected as part of this study. All the data will be securely stored under an alpha-numeric code.

The research data and associated metadata which substantiate published research findings will be made openly available, no later than the date of first online publication or the end of the research project. The open access repositories where the data will be made available are part of the institutional data repository system.

### Timeline

Logistic preparation will take place the two months before the inauguration of the RLWC-2021 tournament. Data collection will take place in October-November 2022 during specific days to be defined. Data analysis, writing up of internal report and potential publications will take place in the following months once the tournament and the data collection has been concluded.

### Patient and public engagement and involvement

PPIE members were not explicitly involved in the development of the research questions, design, recruitment or conduct of this study. However, the development of SNAPPS /eSNAPPS and the design of previous similar studies has benefitted from PPIE input and from many years of clinical and research experience of the authors.

## Dissemination plan

We will disseminate this work by publication of peer reviewed papers in scientific journals. The results will be reported in line with the “Strengthening the Reporting of Observational Studies in Epidemiology (STROBE)” recommendations [17]. We will also present the study at scientific conferences. We will produce infographics to disseminate research findings in an easy to understand format accessible to a wide audience including clinicians, and the general public. The research team will host and provide content to the dedicated SNAPPS website (https://www.snappspfp.com/) and Twitter account (@ProjectSNAPPS).

## Limitations

The biggest limitation for this study is for the tournament to be further postponed or cancelled; the research team is constantly monitoring the news generated by the Tournament organisers, to act to adjust the project timetable. Previous experience of the research team [11] has shown that it is possible to collect data from the expected number of participants within the planned sessions; the eSNAPPS tool allows data collection numbers to be constantly monitored to ensure the targeted number is reached.

## Supporting information

S1 File Demographic questions. (DOCX)

S2 File Quality of life questions. (DOCX)
