# Supplementary material for: The prevalence of patellofemoral pain in the Rugby League World Cup (RLWC) 2021 spectators: A protocol of a cross-sectional study
Source: PLoS One. 2021 Nov 24;16(11):e0260541. doi: 10.1371/journal.pone.0260541 (PMC8612555; doi:10.1371/journal.pone.0260541)
Supplement: S1 File — (DOCX) [file pone.0260541.s001.docx]

Supporting information 1

| Demographic questions |
| --- |
| What is your sex? |
| How old are you? |
| Your answer should be no more than 3 characters long. |
| How tall are you? (m) |
| What is your weight? |
| Where are you from? *(Please type your country of origin)* |
| Where do you live? |
| What is the highest level of education you have completed? *(If currently enrolled, highest degree received.)* |
| Which of the following options better describe your occupation? |
