# Supplementary material for: The prevalence of patellofemoral pain in the Rugby League World Cup (RLWC) 2021 spectators: A protocol of a cross-sectional study
Source: PLoS One. 2021 Nov 24;16(11):e0260541. doi: 10.1371/journal.pone.0260541 (PMC8612555; doi:10.1371/journal.pone.0260541)
Supplement: S2 File — (DOCX) [file pone.0260541.s002.docx]

Supporting information 2

| Quality of life questions | | | | |
| --- | --- | --- | --- | --- |
| (Adapted from the Knee injury and Osteoarthritis Outcome Score (KOOS, LK1.0) questionnaire) | | | | |
| With the following questions we would like to know how much your knee pain affect your quality of life | | | | |
| Never | Monthly | Weekly | Daly | Constantly |
| How often are you aware of your knee problem? | | | | |
| Not at all | Mildly | Moderately | Severely | Totally |
| Have you modified your life style to avoid potentially damaging activities to your knee? | | | | |
| Not at all | Mildly | Moderately | Severely | Extremely |
| How much are you troubled with lack of confidence in your knee? | | | | |
| Not at all | Mildly | Moderately | Severely | Extremely |
| In general, how much difficulty do you have with your knee? | | | | |
| None | Mild | Moderately | Severe | Extreme |
